# Nirmatrelvir and ritonavir combination against COVID‐19 caused by omicron BA.2.2 in the elderly: A single‐center large observational study

**DOI:** 10.1002/iid3.1232

**Published:** 2024-04-05

**Authors:** Can Chen, Ranyi Li, Shuliang Xing, Lei Cao, Yue Qu, Qianzhou Lv, Xiaoyu Li, Zhangzhang Chen

**Affiliations:** ^1^ Department of Pharmacy, Zhongshan Hospital Fudan University Shanghai China; ^2^ Science and Education Office Shanghai Geriatric Medical Center Shanghai China; ^3^ Medical Administration Office, Zhongshan Hospital Fudan University Shanghai China; ^4^ Department of Infectious Diseases The Alfred Hospital and Monash University Clayton Australia

**Keywords:** COVID‐19, nirmatrelvir–ritonavir, observational study

## Abstract

**Background:**

Since coronavirus 2019 (COVID‐19) swept the world, a variety of novel therapeutic and prevention strategies have been developed, among which nirmatrelvir–ritonavir is highly recommended. We intended to assess the effectiveness and safety of nirmatrelvir–ritonavir in the elderly mild‐to‐moderate COVID‐19 population caused by the omicron BA.2.2 variant in real‐world settings.

**Methods:**

An observational study was conducted retrospectively to review the outcomes of mild‐to‐moderate COVID‐19 patients admitted between April 26 and June 30, 2022. Patients' baseline characteristics were collected and assessed. Participants in the intervention group were administered nirmatrelvir–ritonavir in addition to standard care, whereas those in the control group only received standard care. The primary outcome was the duration between the initial positive reverse‐transcription polymerase chain reaction (RT‐PCR) test and the subsequent conversion to a negative result.

**Results:**

The analysis included 324 patients who were administered nirmatrelvir–ritonavir and an equal number of control patients. The patient characteristics in both groups were evenly matched. The average duration from the initial positive RT‐PCR to negative conversion was similar in both groups (16.2 ± 5.0 vs. 16.1 ± 6.3 days, *p* = .83). Control patients exhibited slower conversion in comparison to patients who received nirmatrelvir–ritonavir treatment within 10 days of symptom onset.

**Conclusions:**

These findings suggest that administering nirmatrelvir–ritonavir within 10 days of symptom onset could potentially reduce the time it takes for SARS‐CoV‐2‐infected patients to negative RT‐PCR results, thereby expanding the current usage guidelines for nirmatrelvir–ritonavir.

## 1 INTRODUCTION

The coronavirus 2019 (COVID‐19) pandemic has spurred researchers to develop novel therapeutic and prevention strategies.^1^, ^2^ Vaccines are effective at preventing severe illness, but the immune response may wane over time, particularly against the backdrop of novel variants. This means clinicians need effective antiviral treatments for severe acute respiratory syndrome‐related coronavirus‐2 (SARS‐CoV‐2) infection.

A number of antiviral molecules, monoclonal antibodies, convalescent plasma and combination therapies were investigated and approved clinically since the beginning of pandemic. In the latest World Health Organization (WHO) COVID‐19 living guideline,^3^ nirmatrelvir–ritonavir, molnupiravir and remdesvir were recommended for non‐severe patients with high risk of hospitalization over other alternative treatment options. Nirmatrelvir–ritonavir was given the strong recommendation for its being most effective medication at reducing risk of hospital admission and death compared to molnupiravir and remdesvir.^4^ Nirmatrelvir–ritonavir stands out as a highly potent medication for combating SARS‐CoV‐2. Nirmatrelvir hinders the replication of viruses by focusing on the primary protease (Mpro) of SARS‐CoV‐2^5^ and exhibits comparable effectiveness against both the original SARS‐CoV‐2 strain and its subsequent variants.^6^, ^7^, ^8^ Ritonavir elevates the plasma concentrations of nirmatrelvir by inhibiting CYP3A‐mediated metabolism.^9^ Therefore, combining nirmatrelvir and ritonavir may maximize the therapeutic benefit.^5^, ^10^ The EPIC‐HR study^11^ demonstrated a reduced likelihood of hospitalization or succumbing to the disease in patients with mild to moderate COVID‐19 who were administered nirmatrelvir–ritonavir. Based on the randomized controlled study, numerous countries have granted emergency use authorization to nirmatrelvir–ritonavir for the management of COVID‐19. Subsequently, a series of real‐world studies^12^, ^13^, ^14^, ^15^, ^16^, ^17^ demonstrated the effectiveness of nirmatrelvir–ritonavir.

Medical service providers in China are responsible for monitoring or treating COVID‐19 patients. From March 2022,^18^ nirmatrelvir–ritonavir has been one of the few treatment choices that were accessible for prescription locally. Previous studies^11^, ^12^, ^13^, ^14^, ^15^, ^16^, ^17^ have shown the benefit of nirmatrelvir–ritonavir in general populations, and research on elderly groups is limited. In China and many other countries such as Australia, the majority of COVID‐19 patients at high risk are elderly individuals who have various underlying comorbidities including hypertension, diabetes, and cardiovascular diseases. The purpose of this study was to evaluate the effectiveness of nirmatrelvir–ritonavir in the elderly population in real‐world scenarios, and provide guidance for clinical and policy decision‐making.

## 2 METHODS

### 2.1 Patients, setting, and human ethical approvals

This retrospective observational study, conducted at the Shanghai Geriatric Medical Center (Meilong Branch of Zhongshan Hospital, Fudan University), examined individuals with COVID‐19 who were hospitalized from April 26 to June 30, 2022. The Shanghai Geriatric Medical Center is a specialized medical facility that admits patients with COVID‐19 in Shanghai for treatment. The omicron sublineage BA.2.2 was the primary cause of COVID‐19 cases reported in Shanghai during this time frame.^19^

The patient's infection severity was categorized into 5 types: no symptom, non‐severe, general, and severe types, based on the Diagnosis and Treatment Guideline for COVID‐19 (Trial Version 9) issued by the China National Health Commission.^18^ The high‐risk group for severe COVID‐19 includes individuals aged 60 years or older who have a medical history of hypertension, cardiovascular diseases, cerebral infarction, chronic liver or kidney diseases, cancers, smoking, or obesity.^20^, ^21^, ^22^, ^23^ This analysis included individuals who were diagnosed with no symptom, non‐severe, general and severe types of COVID‐19 and were deemed to be at a high risk of developing severe illnesses. Patients were excluded if they had one reverse‐transcription polymerase chain reaction (RT‐PCR) test during their hospitalization and the result is negative, a severe COVID‐19 diagnosis upon hospitalization, or insufficient information in the electronic medical record, contraindications to nirmatrelvir–ritonavir, severe renal impairment (estimated glomerular filtration rate [eGFR] <30 mL/min per 1.73 m², undergoing dialysis, or had undergone renal transplantation), or severe liver impairment (cirrhosis, hepatocellular carcinoma, or had undergone liver transplantation).

### 2.2 Baseline characteristics of the patients

Upon hospital admission, patients' baseline characteristics were gathered, including sex, age, clinical classification (i.e., severity of symptoms), underlying health conditions, risk factors of developing severe illness, and immunization record. The number of days from a positive RT‐PCR test to initiation of treatment were calculated. Within 2 days of being admitted to the hospital, respiratory samples were obtained from the upper respiratory tract of patients using a nasopharyngeal swab. The samples underwent testing using a real‐time fluorescence quantitative RT‐PCR method to detect the presence of the SARS‐CoV‐2 virus. The targets for the RT‐PCR assay were open reading frame 1ab (ORF), nucleocapsid protein gene (N gene) and envelope protein gene (E gene). All the targets were recorded with the cycle threshold (Ct) value, which represents the number of cycles required to amplify the viral nucleic acid to a detectable level. A Ct value below 35 for ORF, N gene or E gene was used to define a positive sample of SARS‐CoV‐2.^24^

### 2.3 Intervention and control

All of the patients were provided with standard care, which involved resting in bed, monitoring vital signs, measuring oxygen saturation, conducting routine blood chemistry and urine analysis, examining biochemical indicators such as liver and myocardial enzymes and renal function, assessing coagulation parameters, analyzing arterial blood gas, conducting chest imaging and cytokine detection, and administering oxygen therapy if necessary. Individuals diagnosed with mild‐to‐moderate COVID‐19 and had one of the high risks were administered nirmatrelvir at a dosage of 300 mg alongside ritonavir at a dosage of 100 mg, twice a day, for a duration of 5 days. Alternatively, if patients' eGFR ranged from 30 to 59 mL/min per 1.73 m², the dosage of nirmatrelvir was reduced to 150 mg while ritonavir remained at 100 mg. Patients in the control group only obtained standard care and were not administered nirmatrelvir–ritonavir. Patients in both groups did not receive any other medications specifically for COVID‐19, such as remdesivir or monoclonal antibodies.

### 2.4 Primary outcome

The primary outcome was the duration until conversion, which is defined as the time between the initial positive RT‐PCR test and the second negative RT‐PCR test outcome. From the second day of hospitalization onwards, all patients underwent daily RT‐PCR testing using nasopharyngeal swabs until two consecutive negative results were obtained. According to China's COVID‐19 diagnosis and treatment guideline Trial Version 9, criteria for ending isolation and discharge involve obtaining two consecutive tests result with Ct values of ≥35 for ORF, N, and E genes with an interval of more than 24 h.^20^, ^25^

### 2.5 Secondary outcomes and safety assessment

Secondary outcomes included the proportion of individuals with a negative RT‐PCR test for respiratory SARS‐CoV‐2 on the 15th day after the initial positive RT‐PCR test, in‐hospital morality, incidence of turning critically ill, needs for mechanical ventilation and oxygen therapy. In addition, a safety evaluation of the nirmatrelvir–ritonavir was conducted by examining the medical records of patients to identify potential adverse effects (AEs) such as diarrhea and stomach discomfort. Monitoring for AEs began at the start of treatment and continued until the patient was released from the hospital.

### 2.6 Statistical analyses

Data were expressed as frequencies (percentages) for categorical variables, mean (SD) for continuous normally distributed variables, and median (interquartile range, IQR) for continuous non‐normally distributed variables. Propensity score matching was employed based on age, COVID‐19 type upon admission, comorbidities (including diabetes mellitus, malignancy, stroke, hypertension, cardiovascular diseases, pulmonary diseases), vaccination status, and medications administered during hospitalization such as corticosteroids (prednisone, dexamethasone, hydrocortisone, methylprednisolone), antibiotics (β‐lactams, moxifloxacin, levofloxacin, metronidazole, vancomycin, azithromycin, linezolid, tigecycline, caspofungin, voriconazole), antiviral drugs (entecavir, valacyclovir), Chinese traditional medications (Lianhua Qingwen Granules, Qingkailing Soft Capsules, Jinhua Qinggan Granules, Shufeng Jiedu Capsules), immunomodulator (recombinant human interferon α2b injection, human interleukin‐11 for injection, human Granulocyte colony‐stimulating factor injection, intravenous human immunoglobulin, thymalfasin), and Chinese decoctions in a logistic regression model. The propensity‐score matching utilized a caliper width of 0.2 without replacement. The standardized mean differences (SMDs) were calculated for each covariate between the groups before and following the propensity‐score matching. These differences were considered balanced if the SMD was less than the threshold of 0.1.

For the primary outcome, A Kaplan–Meier curve was drawn after propensity score matching. Hazard ratios (HRs) with 95% (confidence intervals, CIs) between nirmatrelvir–ritonavir users and non‐users were estimated using Cox regression models, adjusted for age, sex, vaccination status, COVID‐19 type upon admission, value of first positive PCR, comorbidities, medication use, before and after propensity score matching. Results were stratified by the patients' age (≤60 years vs. >60 years), clinical classification, and vaccination history (fully vaccinated vs. partially vaccinated) at baseline. Additionally, the subgroup analysis was stratified based on the time interval between the administration of nirmatrelvir–ritonavir and the first positive RT‐PCR test.

All the analyses were performed using software SPSS 26.0 and R 4.2.1. Statistical tests were 2‐side and the statistical significance level set at *p* < .05.

## 3 RESULTS

### 3.1 Demographic characteristics of the patients

**Figure 1:**
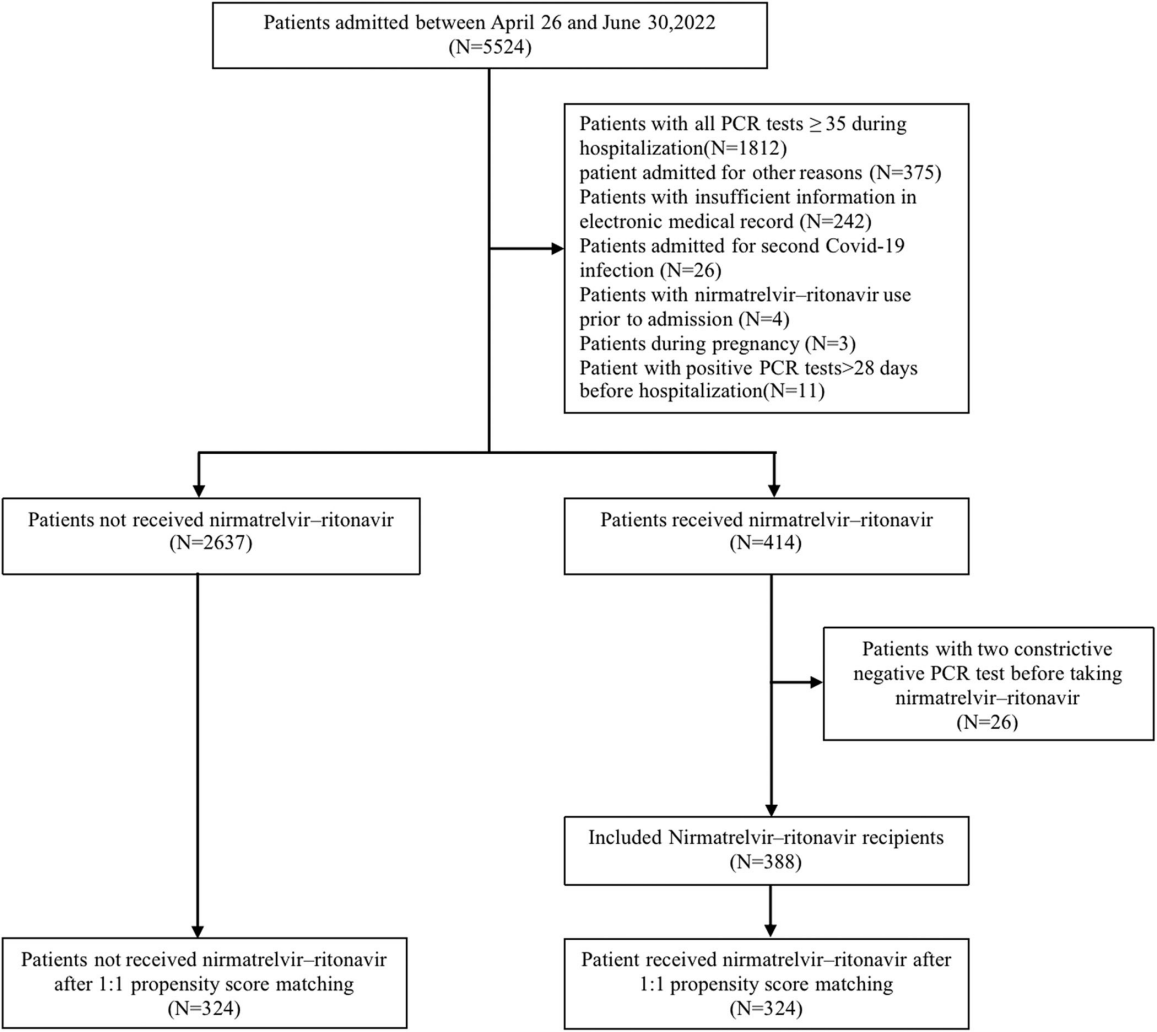
The flow chart of the process used to screen eligible patients. PCR, polymerase chain reaction.

During the designated study period, Shanghai Geriatric Medical Centre admitted a total of 5524 patients who were diagnosed with SARS‐CoV‐2 infections. After removing subjects that did not meet inclusion criteria, the study included 3025 patients. Nirmatrelvir–ritonavir was given to 324 patients (5.8%). The control group consisted of 324 patients from the retrospective cohort, with baseline characteristics used as matching criteria (Figure 1). Following the matching process, patient characteristics were comparable between the nirmatrelvir–ritonavir and control cohorts at baseline, with all SMDs below 0.1, except for RT‐PCR Ct value upon admission (SMD = −0.59), administration of corticosteroids (SMD = 0.11) and administration of antiviral medication (SMD = 0.10). The baseline characteristics of patients treated with or without nirmatrelvir–ritonavir are displayed in Table 1. Overall, less than one‐third of the group were completely immunized, with 194 individuals (29.9%) having been administered at least one dose of the SARS‐CoV‐2 vaccine. Most of the patients were over the age of 65, and the median age was 77 years old (IQR, 67‐88 years). 314 (48.4%) patients were men. 547 (84.4%) were classified as mild cases. The prevalent underlying conditions consisted of individuals aged ≥65 years (530 [81.8%]), hypertension (311 [48.0%]), and diabetes (144 [22.2%]); more than one risk factor was present in 432 (66.7%) patients. There was no significant difference found between the nirmatrelvir–ritonavir and matched control groups.

**Table 1:** Patient baseline clinical characteristics.

	Before 1:1 propensity‐score matching			After 1:1 propensity‐score matching		
	Nirmatrelvir‐ ritonavir (*n* = 388)	Standard care (*n* = 2637)	Standardized mean difference	Standardized mean difference	Nirmatrelvir‐ ritonavir (*n* = 324)	Standard care (*n* = 324)
Age (years), mean (SD)	75.3 (14.4)	54.8 (20.6)	1.43	0.00	74.9 (14.2)	74.9 (14.2)
Male, *n* (%)^a^	189 (48.7)	1399 (53.1)	−0.09	0.05	161 (49.7)	153 (47.2)
Vaccination status, *n* (%)	‐	‐	−0.85	0.07	‐	‐
Vaccine booster completed	18 (4.6)	523 (19.8)	‐	‐	14 (4.3)	9 (2.8)
Fully vaccinated	85 (21.9)	1136 (43.1)	‐	‐	77 (23.8)	85 (26.2)
Partially vaccinated	9 (2.3)	76 (2.9)	‐	‐	7 (2.2)	2 (0.6)
Unvaccinated	253 (65.2)	749 (28.4)	‐	‐	207 (63.9)	190 (58.6)
Unknown	23 (5.9)	153 (5.8)	‐	‐	19 (5.9)	38 (11.7)
RT‐PCR Ct value at admission, mean (SD)^a^	23.0 (5.0)	26.6 (5.4)	−0.71	−0.59	23.1 (5.0)	26.3 (5.7)
Type of COVID‐19 at admission, *N* (%)^b^	‐	‐	0.33	−0.02	‐	‐
No symptom	69 (17.8)	599 (22.7)	‐	‐	63 (19.4)	53 (16.4)
Non‐severe	245 (63.1)	1891 (71.7)	‐	‐	207 (63.9)	224 (69.1)
General	50 (12.9)	91 (3.5)	‐	‐	40 (12.3)	32 (9.9)
Severe	22 (5.7)	27 (1.0)	‐	‐	12 (3.7)	13 (4.0)
Critical	0 (0)	1 (0)	‐	‐	0 (0)	0 (0)
Diabetes mellitus, *n* (%)	89 (22.9)	238 (9.0)	0.33	−0.06	68 (21.0)	76 (23.5)
Malignancy, *n* (%)	22 (5.7)	88 (3.3)	0.10	0.00	21 (6.5)	21 (6.5)
Stroke, *n* (%)	67 (17.3)	154 (5.8)	0.30	0.06	53 (16.4)	46 (14.2)
Hypertension, *n* (%)	191 (49.2)	550 (20.9)	0.57	−0.07	150 (46.3)	161 (49.7)
Heart failure, *n* (%)	21 (5.4)	50 (1.9)	0.16	0.01	15 (4.6)	14 (4.3)
Arrhythmia, *n* (%)	19 (4.9)	46 (1.7)	0.15	0.06	14 (4.3)	10 (3.1)
Coronary atherosclerosis, *n* (%)	63 (16.0)	156 (5.9)	0.27	0.02	46 (14.2)	43 (13.3)
Other heart diseases, *n* (%)	65 (16.8)	159 (6.0)	0.28	0.05	48 (14.8)	42 (13.0)
Pulmonary diseases, *n* (%)	34 (8.8)	62 (2.4)	0.23	−0.02	23 (7.1)	25 (7.7)
Medications used during hospitalization						
Corticosteroids, *n* (%)	43 (11.1)	29 (1.1)	0.32	0.11	24 (7.4)	13 (4.0)
Antibiotics, *n* (%)	123 (31.7)	307 (11.6)	0.43	0.05	84 (25.9)	77 (23.8)
Antiviral, *n* (%)	86 (22.2)	95 (3.6)	0.45	0.10	53 (16.4)	39 (12.0)
Chinese traditional medications, *n* (%)	278 (71.6)	2207 (83.7)	−0.27	−0.02	231 (71.3)	234 (72.2)
Immunomodulator, *n* (%)	107 (27.6)	94 (3.6)	0.54	0.05	56 (17.3)	48 (14.8)
Chinese decoction, *n* (%)	245 (63.1)	975 (37)	0.54	−0.04	202 (62.3)	209 (64.5)

Abbreviations: RT‐PCR, reverse‐transcription polymerase chain reaction; SD, standard deviation.

^a^ These factors were not selected to be covariates in the propensity score matching in this study.

^b^ Non‐severe type: patients experiencing one or multiple mild symptoms such as pyrexia, tussis, pharyngitis, asthenia, myalgia, anosmia and no concomitant pneumonia; general type: similar symptoms to those with mild disease, but they also show radiologic signs of pneumonia; severe type: if patients met any of the following criteria: (1) respiratory rate (RR) ≥ 30 breaths per minute; (2) peripheral oxygen saturation ≤ 93% at rest; (3) PaO_2_/FiO_2_ ≤ 300 mmHg; (4) progressive worsening of clinical symptoms, and lung imaging shows a significant progression >50% within 24‐48 h: Critical type: patients with any of the following conditions: (1) respiratory failure requiring mechanical ventilation; (2) development of shock; (3) concurrent organ failure requiring intensive care.

### 3.2 Application of nirmatrelvir–ritonavir for primary and secondary outcomes

**Figure 2:**
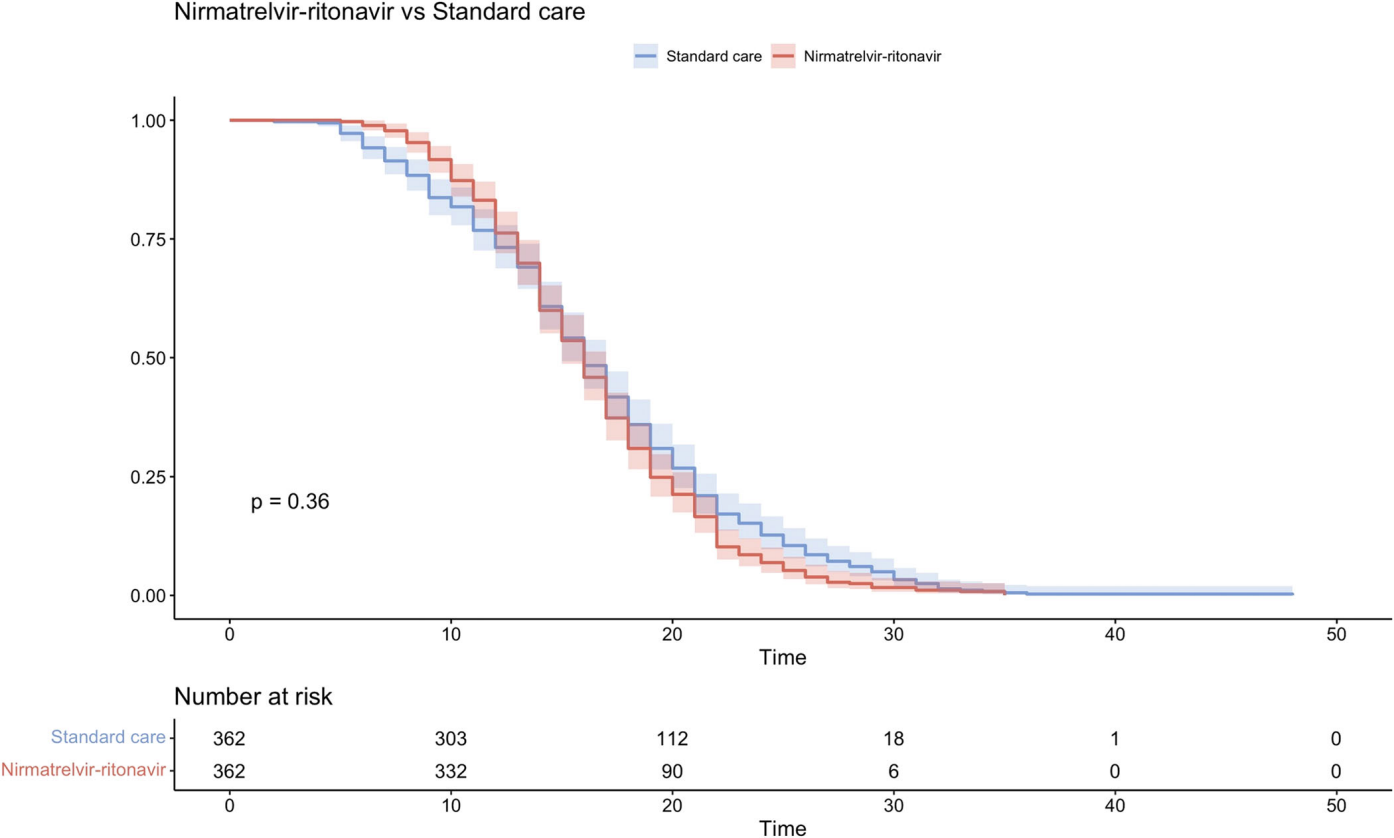
Kaplan–Meier curve of primary outcome for nirmatrelvir–ritonavir recipients versus matched control group.

The mean time from the first positive RT‐PCR result to negative RT‐PCR results was similar in both the treatment and control groups (16.2 ± 5.0 vs. 16.1 ± 6.3 days, *p* = .83, Table 2). Figure 2 displays the Kaplan–Meier curve for the primary outcome, and suggests that nirmatrelvir–ritonavir use was not significantly associated with the shorter time to conversion of RT‐PCR results. Cox regression models showed no association of nirmatrelvir–ritonavir and negative conversion of RT‐PCR value (Table 3).

**Table 2:** Primary and secondary outcomes between patients treated with or without nirmatrelvir–ritonavir.

	Nirmatrelvir–ritonavir (*n* = 324)	Standard care (*n* = 324)	Mean difference or risk ratio (95% CI)	*p* Value
Primary outcome				
Time to viral clearance, mean (SD)	16.19 (5.0)	16.10 (6.3)	0.09 (−0.78 to 0.97)	.83
Secondary outcomes				
The proportion of patients RT‐PCR negative for respiratory SARS‐CoV‐2 at 15 days following symptom onset, *n* (%)	153 (47.2)	162 (50.0)	0.94 (0.81–1.07)	.53
In‐hospital morality, *n* (%)	10 (3.1%)	10 (3.1%)	1.0 (0.37–2.72)	1.00
Incidence of turning critically ill, *n* (%)	6 (1.9)	6 (1.9)	1.0 (0.33–3.07)	1.00
Need for mechanical ventilation, *n* (%)	1 (0.3)	5 (1.5)	0.21 (0.02–1.70)	.21
Need for oxygen therapy, *n* (%)	8 (2.5)	11 (3.4)	0.73 (0.30–1.78)	.64

Abbreviations: CI, confidence interval; RT‐PCR, reverse‐transcription polymerase chain reaction; SARS‐CoV‐2, severe‐acute respiratory syndrome‐related coronavirus‐2; SD, standard deviation.

**Table 3:** Cox regression analysis for COVID‐19 patients received nirmatrelvir–ritonavir.

	Total	HR (95% CI)	*p* Value
Before matching			
Without treatment	2637	Reference	
Treated with nirmatrelvir–ritonavir	388	0.99 (0.87, 1.11)	.834
After matching			
Without treatment	324	Reference	
Treated with nirmatrelvir–ritonavir	324	1.01 (0.86, 1.19)	.905
Treated with nirmatrelvir–ritonavir ≤10 days	266	1.35 (1.10, 1.66)	.004

*Note*: Adjusted by age, sex, vaccination status, Covid‐19 type at admission, first positive PCR value, comorbidities (diabetes, malignancy, stroke, hypertension, heart failure, arrhythmia, coronary artery disease, pulmonary diseases), medication use (corticosteroids, antibiotics, antiviral, Chinese medicine, immunomodulator, Chinese decoction).

Abbreviations: CI, confidence intervals; HR, hazard ratio.

**Figure 3:**
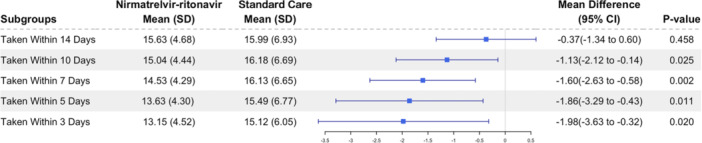
Subgroup analysis of the mean difference of time to viral clearance for patients with different nirmatrelvir–ritonavir initiation time versus matched control group.

Following the initial RT‐PCR confirmation, 92% of was administered within 14 days, while 82% and 64% were taken within 10 and 7 days respectively. Hence, the subgroup analysis was further stratified based on the time interval between the administration of nirmatrelvir–ritonavir and the first positive RT‐PCR test. Patients who received nirmatrelvir–ritonavir within 10 days of experiencing symptoms demonstrated quicker conversion of RT‐PCR results compared to the control group that was matched (Figure 3). Cox regression analysis also demonstrated that patients treated with nirmatrelvir–ritonavir ≤10 days was associated with a shorter negative conversion time of RT‐PCR value (Table 3).

**Figure 4:**
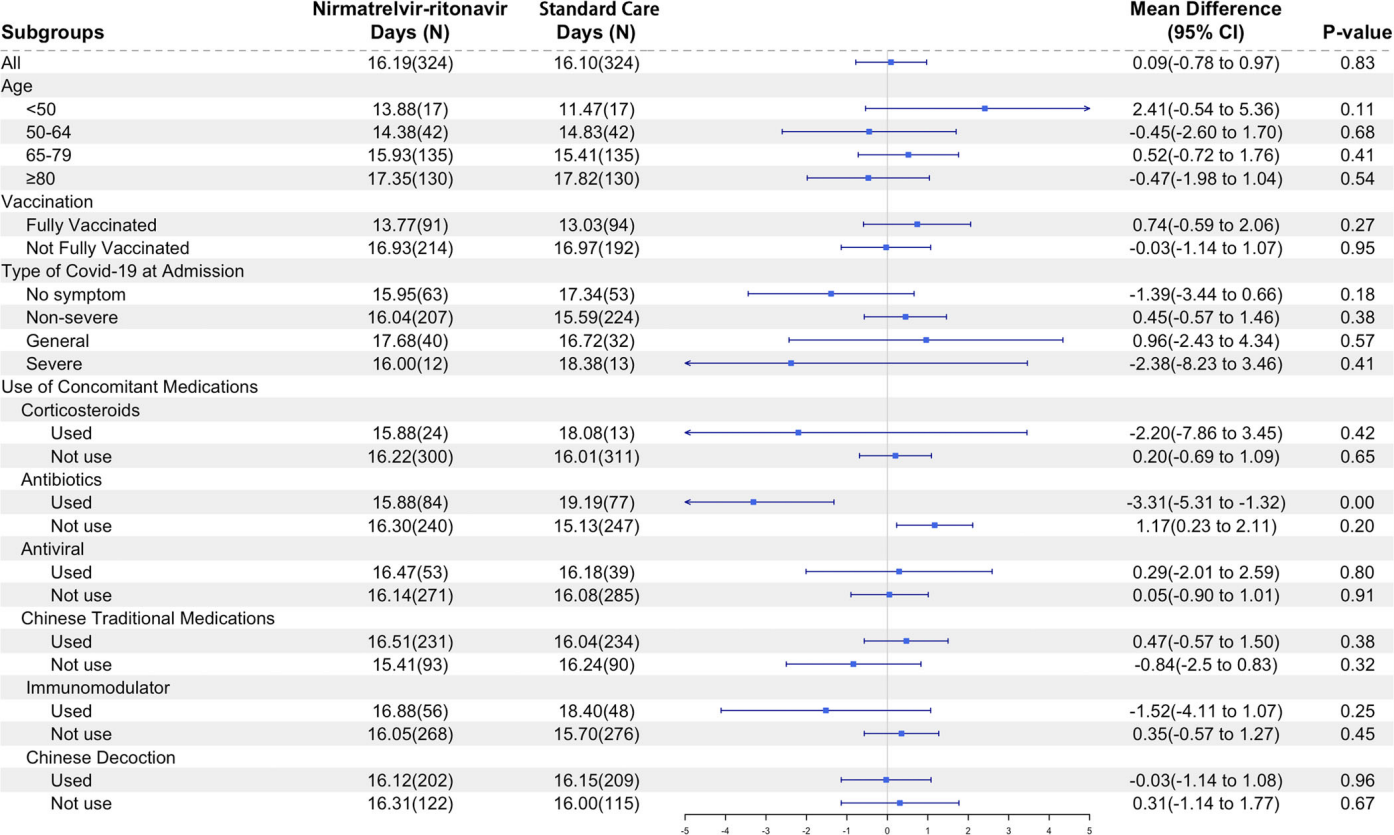
Subgroup analysis of the mean difference of time to polymerase chain reaction (PCR) negative for nirmatrelvir–ritonavir recipients versus matched control group. CI, confidence interval; SD, standard deviation.

Stratified analyses are shown in Figure 4. No significant correlation was observed between the time to conversion of RT‐PCR results and patients' age, clinical classification, concurrent medication usage, or prior vaccination in the analysis of individuals treated with nirmatrelvir–ritonavir, except in the case of the usage of antibiotics (Figure 4).

**Figure 5:**
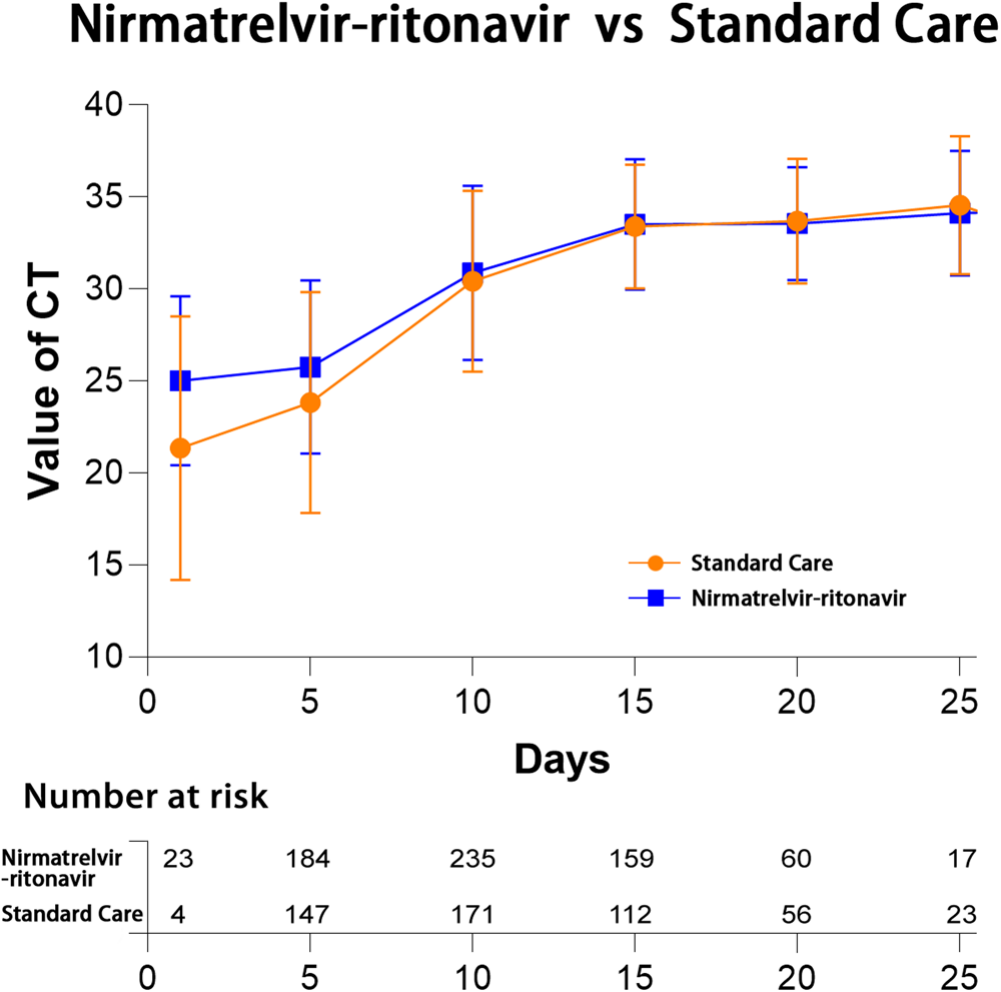
Change of reverse‐transcription polymerase chain reaction (RT‐PCR) Ct value for nirmatrelvir–ritonavir recipients versus matched control group.

No significant difference was found between the two groups in the percentage of patients who tested negative for respiratory SARS‐CoV‐2 using RT‐PCR 15 days after the onset of symptoms (47.2% vs. 50.0%, *p* = .53) (Figure 5). Additionally, the two groups did not differ significantly with respect to the progression to more severe conditions, including in‐hospital mortality (3.1% vs. 3.1%, *p* = 1.00), turning to critically ill (1.9% vs. 1.9%, *p* = 1.00), need for mechanical ventilation (0.3% vs. 1.5%, *p* = .21), and the need for oxygen therapy (2.5% vs. 3.4%, *p* = .64).

### 3.3 Evaluation of the safety of nirmatrelvir–ritonavir in COVID patients at high risk

Among patients treated with nirmatrelvir–ritonavir, 64 patients reported the AEs. Out of these patients, 19 (29.7%) encountered at least one form of AEs, with gastrointestinal disorders (21.8%) being the prevailing side effects. The detailed information is found in Table 4.

**Table 4:** The occurrence of adverse effects in treatment group.

	Nirmatrelvir–ritonavir (*n* = 64)
Gastrointestinal disorders	
Taste disturbances	8
Decreased appetite	4
Dry mouth	1
Vomiting	1
Immune system disorders	
Skin rash	2
General disorders	
Fatigue	1
Cardiac disorders	
Palpitations	2

## 4 DISCUSSION

In May 2023, the global health emergency of COVID‐19 was officially terminated by the WHO. The attention for COVID‐19 care and treatment has now been redirected towards the vulnerable population who are at a higher risk of developing more serious COVID‐19‐associated illnesses. In the midst of a widespread omicron outbreak and high vaccination rates, our study revealed that the utilization of nirmatrelvir–ritonavir did not result in a more rapid elimination of respiratory SARS‐CoV‐2 in patients at high risk.

Interestingly, the examination centered on individuals who received nirmatrelvir–ritonavir treatment within 10 days after first positive RT‐PCR test. It revealed a faster conversion from positive to negative RT‐PCR tests when compared to untreated patients. The study also found that the shorter the time between the first positive RT‐PCR test and the administration of nirmatrelvir–ritonavir, the faster the conversion to RT‐PCR negativity. This indicates that the timing of nirmatrelvir–ritonavir therapy initiation following the onset of symptoms is a crucial indicator in achieving rapid elimination of the virus. Our findings align with a recent study^15^ which indicated that the administration of nirmatrelvir–ritonavir may accelerate the conversion of negative RT‐PCR respiratory SARS‐CoV‐2. Additionally, our study expands upon the findings of the EPIC‐HR study,^11^ which included unvaccinated and nonhospitalized adults from various racial backgrounds. In contrast, our study specifically examined Asian adult patients at high risk who were hospitalized, fully or partially vaccinated and generally “older.”

RT‐PCR testing has been utilized to detect SARS‐CoV‐2 in the upper respiratory tract.^26^ The Ct values of RT‐PCR represent the number of amplification cycles needed for the target gene to exceed a threshold level, potentially offering semiquantitative or indirect measurements of viral load.^27^ Previous studies have analyzed the temporal trends in Ct values over the course of a SARS‐CoV‐2 infection. The lowest Ct values (indicating a higher concentration of viral RNA) are observed shortly after the onset of symptoms and are significantly correlated with the time elapsed since onset. Higher Ct values may be linked to better outcomes in COVID‐19 individuals, decreased possibility of progression to severe illness, decreased disease severity, decreased mortality, and absence of biochemical and hematological markers.^27^ The RT‐PCR Ct value was frequently used as an alternative measure of viral load, serving as a measure of infectivieness. According to this research, the administration of nirmatrelvir–ritonavir within 10 days of the symptom onset resulted in a quicker rise in RT‐PCR Ct value, thus reducing the duration of isolation. It is reported that early administration of nirmatrelvir–ritonavir in patients could lead to a quicker cessation of virus shedding, thereby reducing the risk of disease transmission and expediting recovery from COVID‐19.^27^

Previous study noted that SARS‐CoV‐2 viral replication at multiple sites outside the respiratory tract during the first 2 weeks after the onset of symptoms.^28^ The peak viral load of SARS‐CoV‐2 typically occurred around the time of symptoms onset, followed by a gradual decline.^29^ In severely ill patients, viral replication can continue for several months.^28^ Based on the findings mentioned above, it should be considered that patients with COVID‐19 more than 5 days post‐onset and/or severe illness are highly probable to derive benefits from nirmatrelvir–ritonavir therapy. This is due to the continued presence of the virus in the body, particularly in the respiratory system, among patients in the early (within 14 days after symptoms onset), intermediate (15–30 days), and late (31 days or longer) COVID‐19 patients. At this time, nirmatrelvir–ritonavir is given to clear the virus, which theoretically benefits the patient's recovery and may even prevent their death. Furthermore, owing to the scarcity of COVID‐19 healthcare facilities in Shanghai during the initial 6 months of 2022, a number of individuals testing positive for PCR were unable to receive immediate admission following infection, resulting in a disease duration exceeding 5 days upon admission. Therefore, many patients admitted in Shanghai Geriatric Medical Center did not receive nirmatrelvir–ritonavir until RT‐PCR was positive or symptoms occurred more than 5 days. The current research validated that administering nirmatrelvir–ritonavir within 5–10 days of symptom onset or a positive RT‐PCR test could expedite the conversion to a negative result.

The limitation of the current study cannot be ignored. The study is a single‐centered retrospective observational study with potential residual confounding and selection bias. Because the study was conducted retrospectively, a detailed analysis of adverse events and rebound were not performed, as the data in medical records regarding these topics were sometimes inconsistent.

## CONCLUSION

To summarize, the use of nirmatrelvir–ritonavir within 10 days of symptom onset or a positive RT‐PCR result is linked to a reduced duration of RT‐PCR conversion in high‐risk patients infected with SARS‐CoV‐2. This expands the potential use of nirmatrelvir–ritonavir beyond its labeled indications.

## AUTHOR CONTRIBUTIONS

**Can Chen**: Investigation; methodology; writing—original draft; writing—review and editing. **Ranyi Li**: Data curation; formal analysis; software; writing—review and editing. **Shuliang Xing**: Project administration; resources; supervision. **Lei Cao**: Project administration; resources; supervision. **Yue Qu**: Methodology; writing—review and editing. **Qianzhou Lv**: Supervision. **Xiaoyu Li**: Conceptualization; data curation; funding acquisition; writing—review and editing. **Zhangzhang Chen**: Conceptualization; data curation; formal analysis; writing—review and editing.

## CONFLICT OF INTEREST STATEMENT

The authors declare no conflicts of interest.

## ETHICS STATEMENT

This study adhered to STROBE guidelines and received approval from the ethics committees of Zhongshan Hospital, Fudan University (approval number B2022‐470R).

## PATIENT CONSENT STATEMENT

Due to the exceptional circumstances of the COVID‐19 outbreak, this retrospective study utilizing deidentified information did not necessitate individual patient‐informed consent.

## Supporting information

Supporting information.

## Data Availability

The data that support the findings of this study are available from the corresponding author upon reasonable request.
